# Supplementary material for: In Situ Electrochemical Formation of Oxo-Functionalized Graphene on Glassy Carbon Electrode with Chemical Fouling Recovery and Antibiofouling Properties for Electrochemical Sensing of Reduced Glutathione
Source: Antioxidants (Basel). 2022 Dec 21;12(1):8. doi: 10.3390/antiox12010008 (PMC9854563; doi:10.3390/antiox12010008)
Supplement: Supplementary file 1 [file antioxidants-12-00008-s001.zip › antioxidants-2074625-supplementary.pdf]

Supplementary materials for

In situ electrochemical formation of oxo-functionalized graphene on glassy carbon electrode with chemical fouling recovery and antibiofouling properties for electrochemical sensing of reduced glutathione

Chunying Xu †, Gang Li †, Liju Gan, and Baiqing Yuan *

School of Chemistry and Materials Science, Ludong University, Yantai 264025, Shandong, China

*** Corresponding author.**

E-mail: bqyuan@ldu.edu.cn; baiqingyuan1981@126.com

† These authors contributed equally to this work.

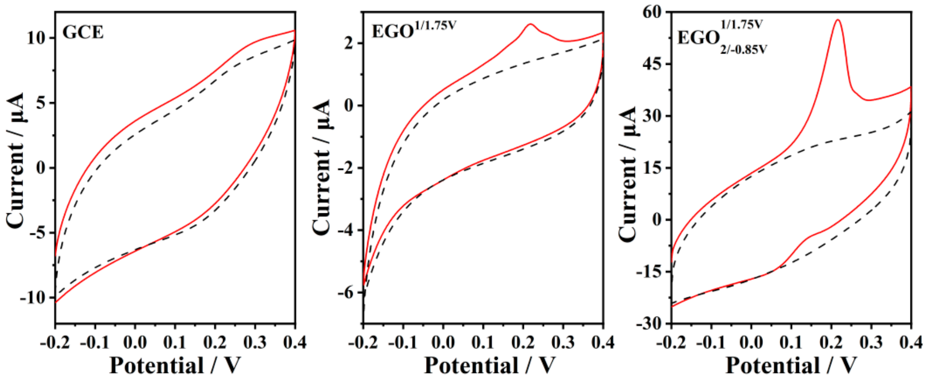

Figure S1 The CVs of GCE, $EGO^{1/1.75V}$, and $EGO^{1/1.75V}_{2/-0.85V}$ in the presence (solid line) and absence (dotted line) of 5 mM GSH in pH 4.5 PBS solution.

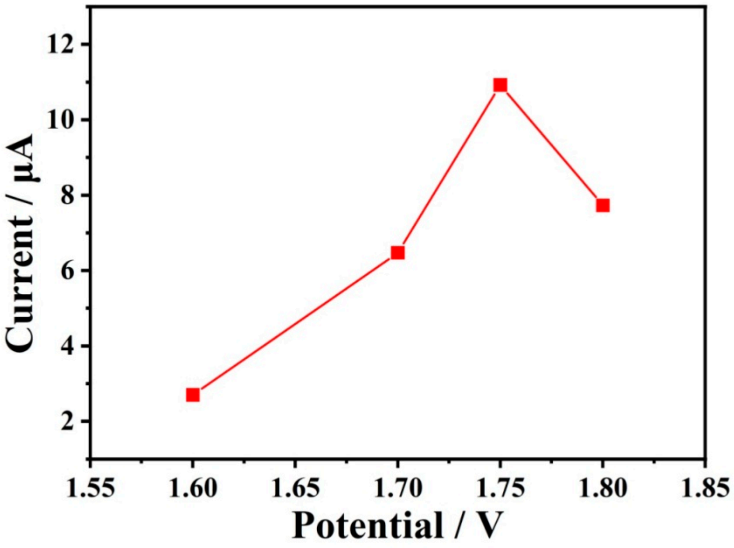

Figure S2 The effect of oxidation potential for $EGO^{1/potential}_{2/-0.85V}$ on the oxidation current of GSH. Buffer: 0.1 M pH 5.0 PBS; Oxidation time: 200 s; Reduction potential; -0.85 V; Reduction time: 500s.

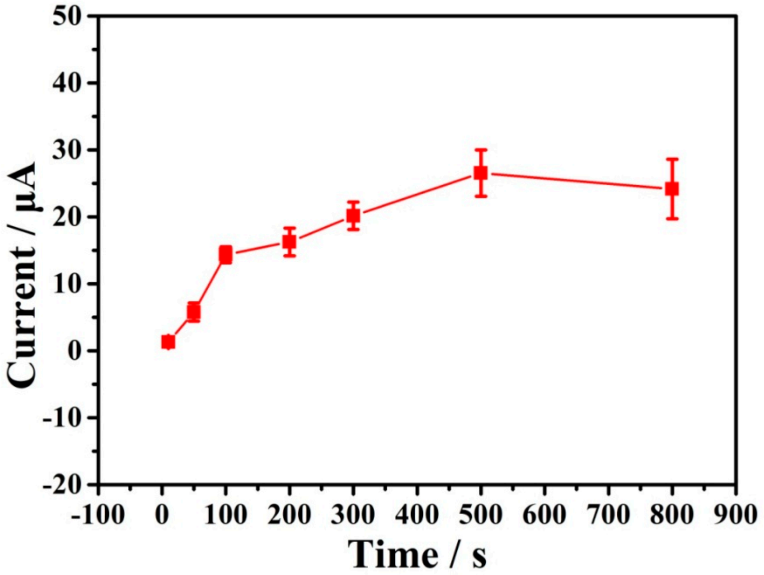

Figure S3 The effect of oxidation time for $EGO_{2/-0.85V}^{1.75V}$ on the oxidation current of GSH. Buffer: 0.1 M pH 5.0 PBS; Reduction potential; -0.85 V; Reduction time: 500s.

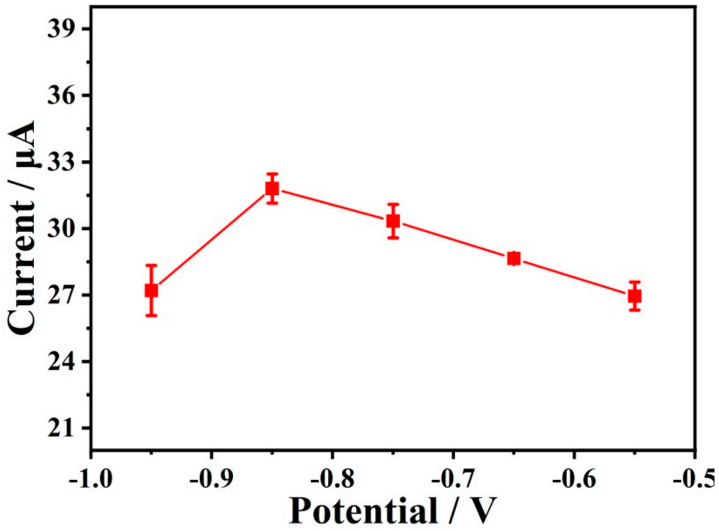

Figure S4 The effect of reduction potential for $EGO_{2/potential}^{1.75V}$ on the oxidation current of GSH. Buffer: 0.1 M pH 5.0 PBS; Oxidation potential: 1.75 V; Oxidation time: 500 s.

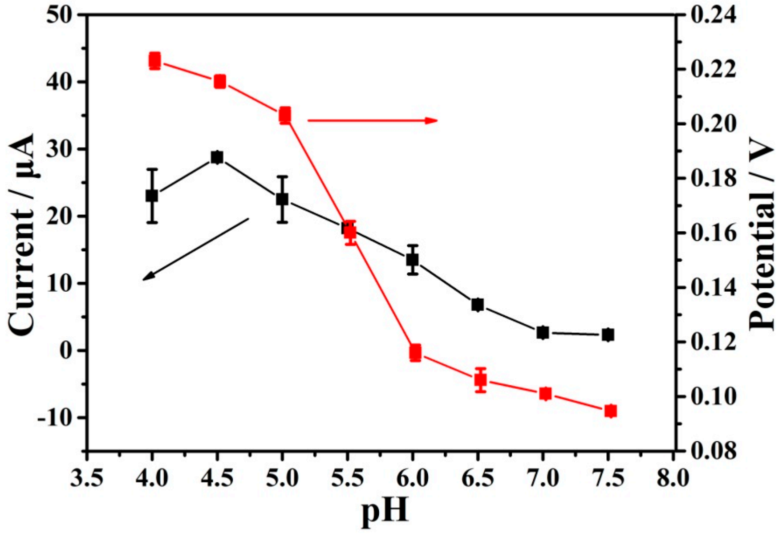

Figure S5 The effect of pH of solution on the CV oxidation current and oxidation potential of GSH at $EGO_{2/-0.85v}^{1/1.75V}$. Oxidation time: 500 s; Reduction potential; -0.85 V; Reduction time: 500s.

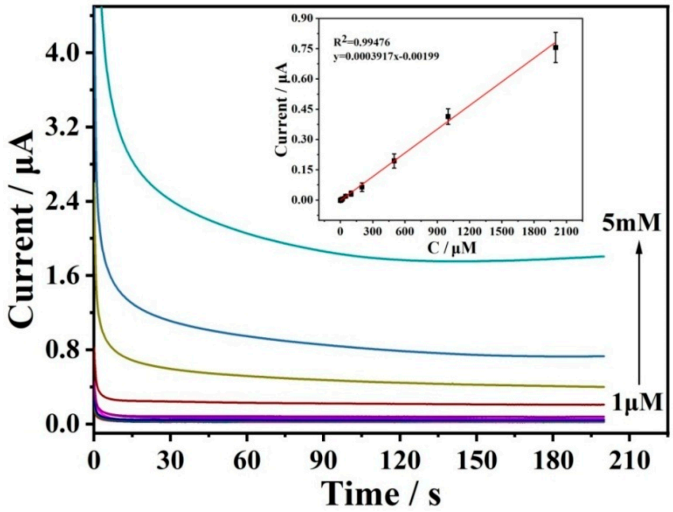

Figure S6 The amperometric responses of $EGO_{2/-0.85v}^{1/1.75V}$ to various concentrations of GSH in a static 0.1 M PBS (pH=4.0) at 0.2 V. (Inset: the corresponding calibration plot.)

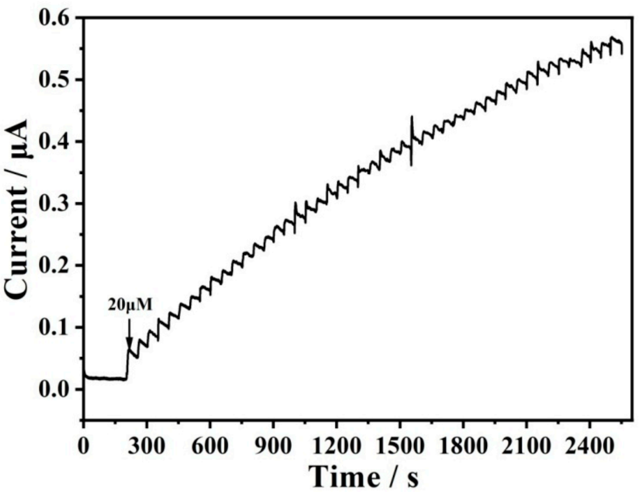

Figure S7 Repeated measurements of GSH using the same electrode

Table S1 Fitted parameters for Raman spectra

Material	D*			D			D’’			G			D’		
	X_c(cm^{-1})	W(cm^{-1})	A(%)	X_c(cm^{-1})	W(cm^{-1})	A(%)	X_c(cm^{-1})	W(cm^{-1})	A(%)	X_c(cm^{-1})	W(cm^{-1})	A(%)	X_c(cm^{-1})	W(cm^{-1})	A(%)
**GCE**	1249	263	10.3	1348	70	35	1495	184.7	10.7	1592	70.2	16.7	1618	48	2.9
$\mathbf{EGO^{1/1.75V}}$	1134	194	4.8	1348	135.2	43.8	1510	152.7	7.2	1578	85.9	16.6	1610	53.2	6.8
$\mathbf{EGO^{1/1.75V}_{2/-0.85V}}$	1169	273.2	7.6	1344	113.7	44.5	1547	138.5	13.8	1600	68.2	10.7	1605	43.8	3.4

Table S2 Fitted parameters for Raman spectra

Material	G*			2D			D+D’			2D’		
	X_c(cm^{-1})	W(cm^{-1})	A(%)	X_c(cm^{-1})	W(cm^{-1})	A(%)	X_c(cm^{-1})	W(cm^{-1})	A(%)	X_c(cm^{-1})	W(cm^{-1})	A(%)
**GCE**	2488	156.7	0.8	2697	124.9	15.4	2934	131	6.7	3208	136.8	1.2
$\mathbf{EGO^{1/1.75V}}$	2545	367.2	1.3	2684	266.6	9.6	2930	195.5	8.4	3188	133.6	1.4
$\mathbf{EGO^{1/1.75V}_{2/-0.85V}}$	2621	149.4	0.7	2696	220.1	11	2926	176.8	5.8	3185	128.6	2.1

Table S3 Calculated ratio for components

Material	I_D/I_G	I_{D*}/I_G	A_D/A_G	$A_{D'}/A_G$
**GCE**	2.04	0.18	2.1	0.17
$\mathbf{EGO^{1/1.75V}}$	1.7	0.11	2.6	0.41
$\mathbf{EGO^{1/1.75V}_{2/-0.85V}}$	2.0	0.15	4.1	0.32

Table S4 Fitted parameters for C1s spectra

Electrodes		C-C/C=C	C-O	C=O	O-C=O
GCE	binding energy	284.54	285.8	287	
	Content%	68.9	26.3	4.7	
$\mathbf{EGO^{1/1.75V}}$	binding energy	284.81	285.8	287.02	288.44
	Content%	44.6	18.3	17.3	19.8
$\mathbf{EGO_{2/-0.85V}^{1/1.75V}}$	binding energy	284.71	285.8	287	288.48
	Content%	56.5	31.5	0.3	11.7

Table S5 Fitted parameters for O1s spectra

Electrodes		O-C=O	C=O	C-OH	C-O-C	chemisorbed oxygen and/or water
GCE	binding energy	530.94	531.68	532.6	533.41	
	Content%	1.8	19.7	49.9	28.5	
$\mathbf{EGO^{1/1.75V}}$	binding energy	530.93	531.68	532.72	533.4	534.95
	Content%	12.4	26.6	32.4	21.8	6.8
$\mathbf{EGO^{1/1.75V}_{2/-0.85V}}$	binding energy	530.93	531.4	532.61	533.6	535
	Content%	9.0	21	39.4	17.7	12.9

Table S6. EIS fitted parameters for different electrodes.

Electrodes	R1	R2	W1-R	W1-T	W1-P	CPE1-T	CPE1-P
**GCE**	110	429.5	7734	51.81	0.47483	2.2257E-6	0.86256
**EGO_1**	102	5375	8193	27.1	0.58542	9.7352E-6	0.88546
**EGO_2**	105.7	194.1	11731	80.81	0.49671	0.00011586	0.8512
